# Modulatory Role of Biochar Properties and Environmental Risk of Heavy Metals by Co-Pyrolysis of Fenton Sludge and Biochemical Sludge

**DOI:** 10.3390/toxics12010057

**Published:** 2024-01-11

**Authors:** Yujian Li, Mengen Kang, Yuting Wang, Xue Bai, Zhengfang Ye

**Affiliations:** 1 Key Laboratory of Integrated Regulation and Resource Development on Shallow Lake of Ministry of Education, College of Environment, Hohai University, Nanjing 210098, China; 2 Yangtze Institute for Conservation and Development, Hohai University, Nanjing 210098, China; 3 Key Laboratory of Water and Sediment Sciences, Department of Environmental Engineering, Peking University, Ministry of Education, Beijing 100871, China

**Keywords:** Fenton sludge, biochemical sludge, co-pyrolysis, heavy metals, environmental risk

## Abstract

Recent studies have reported that Fenton sludge and biochemical sludge contain high concentrations of toxic substances and heavy metals (HMs), whereas improper treatment can pose serious threats to environmental safety. Pyrolysis is considered an efficient technology to replace conventional sludge treatment. This study investigated the pyrolysis and kinetic processes of Fenton sludge and biochemical sludge, revealed the physicochemical properties of sludge biochar, and highlighted the role of co-pyrolysis in sludge immobilization of HMs and environmental risks. Results showed that Fenton sludge and biochemical sludge underwent three stages of weight loss during individual pyrolysis and co-pyrolysis, especially co-pyrolysis, which increased the rate of sludge pyrolysis and reduced the decomposition temperature. The kinetic reaction indicated that the activation energies of Fenton sludge, biochemical sludge, and mixed sludge were 11.59 kJ/mol, 8.50 kJ/mol, and 7.11 kJ/mol, respectively. Notably, co-pyrolysis reduced the activation energy of reactions and changed the specific surface area and functional group properties of the biochar produced from sludge. Meanwhile, co-pyrolysis effectively immobilized Cu, Pb, and Zn, increased the proportion of metals in oxidizable and residual states, and mitigated the environmental risks of HMs in sludge. This study provided new insights into the co-pyrolysis properties of sludge biochar and the risk assessment of HMs.

## 1. Introduction

Following the rapid technological advancements in wastewater treatment, the production of sludge as a by-product of the process has steadily increased [1]. Due to the complex nature of wastewater, the resulting sludge typically contains high levels of organic matter and nutrients such as nitrogen, phosphorus, and potassium [2], as well as contaminants such as pathogens and heavy metals (HMs) [3]. Reducing the bioavailability of HMs in sludge has become a serious challenge in current research because HMs are highly toxic and have negative effects on humans and the environment [4]. Fenton sludge and biochemical sludge are two common types of sludge in chemical plants [5]. Wu et al. revealed the mechanism of the effect of Fenton sludge on methanogenic activity by incorporating Fenton sludge into an anaerobic digestion system [6]. Chi et al. achieved sludge reduction and nutrient removal by lysing biochemical sludge and returning it to the biochemical tanks [7]. Biochemical sludge is more difficult to treat than municipal sludge due to the high levels of pathogens and HMs in chemical wastewater, as well as the higher levels of organic matter [8]. The proper management of Fenton sludge as hazardous waste should be delegated to organizations possessing the necessary qualifications [9]. This not only increases the cost of sludge treatment but also wastes a large amount of the iron and aluminum resources present in Fenton sludge [10,11]. Based on this, this study proposes a method of co-pyrolysis of Fenton sludge and biochemical sludge to achieve simultaneous treatment of sludge by-products generated by the chemical industry. This not only achieves the immobilization of HMs in sludge but also increases the potential of sludge resource utilization.

As a modern method of sludge treatment, pyrolysis is an efficient approach to sludge reduction, stabilization, mitigation, and recycling [12,13]. Studies have demonstrated the effectiveness of sludge pyrolysis in reducing sludge volume and using organic matter to produce sludge biochar at minimal cost [14]. Concurrently, pyrolysis of sludge has been shown to efficiently immobilize HMs [15], thereby lowering their bioavailability in sludge [16] and facilitating the reuse of sludge products [17]. Since the co-pyrolysis process of sludge produces catalytic and synergistic effects, the resulting co-pyrolysis biochar has found diverse applications in environmental treatments, including soil improvement [18,19], pollutant adsorption [20], and catalytic reactions [21]. Currently, studies confirm that co-pyrolysis of biomass or chemical additives with sludge enhances the stability of HMs and reduces the toxicity of sludge biochar [22]. However, there is limited research on immobilizing HMs by co-pyrolyzing two sludges with different properties. Chen et al. [23] conducted microwave pyrolysis on sewage sludge and electroplating sludge with elevated levels of HMs. The results of the study showed that the immobilization rate of HMs in pyrolytic biochar produced from electroplating sludge was less than 75%, whereas the immobilization rate of HMs in co-pyrolytic biochar increased to 98.00%. The study found that both organic carbon and inorganic minerals present in sewage sludge play a significant role in immobilizing HMs through physical and chemical actions.

This study examined the pyrolysis characteristics of Fenton sludge and biochemical sludge, clarified the kinetic process of individual pyrolysis and co-pyrolysis of sludge, and analyzed the various indicators that produced sludge biochar. The main objectives of this study were to (1) Investigate the pyrolysis process and properties of sludge during diverse heating rates in both individual pyrolysis and co-pyrolysis. (2) Clarify the kinetic reactions of Fenton sludge, biochemical sludge, and mixed sludge with an analysis of activation energy and reactivity. (3) Reveal the physicochemical properties and structural composition of pyrolytic biochar in different sludges. (4) Elucidate the effect of co-pyrolysis on HM immobilization in sludge and assess potential environmental risks.

## 2. Materials and Methods

### 2.1. Experimental Sludge and Properties

As a representative nitroaromatic compound with high chemical stability and high biological toxicity, *p*-nitrophenol (PNP) is derived from a variety of industries, including explosives, dyes, and leather [24]. The Fenton sludge in this study was obtained by simulating the PNP wastewater treated with the Fenton and flocculation process of a chemical group in Gansu, China, and the biochemical sludge was sourced from the Nanjing wastewater plant in China. This wastewater treatment plant utilizes a secondary treatment process that includes screening, grit removal, primary sedimentation, and activated sludge. The sludge samples were collected from the wastewater treatment plant after mechanical dewatering. The Fenton sludge and biochemical sludge were dried and sieved in a well-ventilated area to avoid spoiling the samples.

### 2.2. Individual Sludge Pyrolysis

The Fenton sludge and biochemical sludge were dried in an oven at 80 °C for 24 h and then retained. Both types of sludge were individually ground in a mortar and subsequently sieved through a 100-mesh (0.15 mm) sieve. The Fenton sludge and biochemical sludge were weighed at 10 ± 0.1 mg each and then transferred to platinum crucibles. Then, the samples were examined with a thermogravimetric analyzer (TGA) (TGA8000, PerkinElmer, Waltham, MA, USA) and heated from room temperature to 800 °C at a nitrogen flow rate of 30 mL/min with ramping rates of 10, 20, and 30 °C/min. Thermogravimetry/Derived Thermogravimetry (TG/DTG) was used to record the change in sludge weight with increasing temperature.

### 2.3. Co-Pyrolysis of Mixed Sludge

The two types of sludge were ground in a mortar at a weight ratio of 1:1 to obtain a mixed sludge, then sieved through a 100-mesh (0.15 mm) sieve. The mixed sludge was weighed at 10 ± 0.1 mg and analyzed through TGA testing, following the same method as the individual pyrolysis of the sludge. The heating rates and TG/DTG were recorded according to the details provided above.

### 2.4. Calculation of Activation Energy

The theory of thermodynamic analysis is well developed for the study of the formation process and substance growth kinetics. The study determined the reaction kinetic parameters and analyzed the mechanism by measuring TG curves at various pyrolysis rates. These findings provided crucial insights into the experimental process of pyrolysis and the resulting physical properties, phases, and shapes of the products [25]. The fundamental equation for the kinetics of non-homogeneous solid-state thermal decomposition is illustrated in Equation (1) [26].

(1) $$\frac{d\alpha }{dt}=k\left(T\right)f\left(\alpha \right)=Aexp\left(\frac{{-E}_{\alpha }}{RT}\right)f\left(\alpha \right)$$

where *t* stands for the time; *T* stands for reaction temperature; *A* stands for pre-exponential factor; *k*(*T*) stands for the rate constant related to temperature; *f*(*α*) stands for the dependence of the conversion rate *α* with respect to the response model; *E_α_* stands for the apparent activation energy at the conversion rate *α*; *R* stands for the universal gas constant and is 8.314 J/(mol·K).

When the rate of heating, *β* = *dT*/*dt*, is introduced, Equation (1) is transformed into Equation (2).

(2) $$g\left(\alpha \right)=\beta \frac{d\alpha }{dT}=Aexp\left(\frac{-E_{\alpha }}{RT}\right)f\left(\alpha \right)$$

This study utilized the Flynn-Wall-Ozawa (FWO) method to determine and analyze the activation energy for the pyrolysis of Fenton sludge, biochemical sludge, and mixed sludge. The FWO method relies on the Doyle equation and subsequently converts Equation (2) to Equation (3).

(3) $$ln\beta =ln\left(\frac{0.0048AE_{\alpha }}{Rg\left(\alpha \right)}\right)-1.0516\frac{E_{\alpha }}{RT}$$

where *β* stands for the rate at which temperature increases; *T* stands for reaction temperature; *A* stands for pre-exponential factor. *E_α_* stands for the apparent activation energy at the conversion rate *α*; *g*(*α*) stands for the integral form of the mechanism function; *R* stands for the universal gas constant and is 8.314 J/(mol·K).

When the conversion rate *α* is determined, *g*(*α*) is a constant value. A linear relationship with a slope of −1.0516 *E_α_*/*R* was established by plotting *1*/*T* on the x-axis and *logβ* on the y-axis to determine *E_α_* values associated with different conversion rate *α*. The average activation energy *E* was then calculated.

### 2.5. Preparation of Sludge Biochar

The designated amounts of Fenton, biochemical, and mixed sludge (referred to as m_1_) were spread evenly on a porcelain tray individually. The tray was covered and placed in the tube furnace. The furnace was heated from room temperature to 800 °C at a heating rate of 5 °C/min, then held at that temperature for 1 h in a steady nitrogen atmosphere. The sludge biochar was obtained by cooling it to room temperature in a tube furnace and later dried to a constant weight for storage (referred to as m_2_). The biochar produced was named FBC-800, BBC-800, and MBC-800. The yield of the biochar was calculated by Equation (4).

(4) $$Y=\left(\frac{m_{2}}{m_{1}}\right)\times 100\%$$

### 2.6. Characterization and Analysis of Sludge Biochar

The pH of sludge biochar was determined with reference to the “Determination of pH value of charcoal activated carbon test method” (GB/T12496.7-1999). The contents of C and H were analyzed using an organic elemental analyzer (UNICUBE, Elementar, Frankfurt, Germany), while the Si, Fe, and Al levels were determined through an Inductively Coupled Plasma Emission Spectrometer (ICP-OES) (Avio 200, PerkinElmer, Waltham, MA, USA). Furthermore, the specific surface area of the biochar was examined using a fully automated specific surface and porosity analyzer (ASAP 2460, Micromeritics, Atlanta, GA, USA). The microscopic morphology was visualized by scanning electron microscopy (SEM) (FEI Scios 2 HiVac, Thermo, Waltham, MA, USA). The physical phase composition and functional group structure were analyzed by X-ray diffractometer (XPS) (Smartlab 9KW, Rigaku, Akishima-shi, Japan) and Fourier transform infrared microscopy (FTIR) (Spotlight 200i, PerkinElmer, Llantrisant, UK), respectively.

### 2.7. Immobilization and Risk Assessment of HMs

The total content of Cu, Pb, and Zn in the sludge biochar, as well as their corresponding content in different forms, were determined by ICP-OES. To ensure data accuracy, three parallel groups were established for each of the three types of sludge biochar. The immobilization rate *R* of HMs in sludge was computed using Equation (5).

(5) $$R=\frac{C_{2}\times Y}{C_{1}}$$

where *C*_1_ stands for the content of HMs before pyrolysis; *Y* stands for yield; and *C*_2_ stands for the content of HMs after pyrolysis.

The various types of HMs in sludge biochar were extracted by the sequential extraction method as described by Li et al. [27]. The HMs in sludge biochar consisted of four main forms, including F1: acid-soluble fraction, F2: reducible fraction, F3: oxidizable fraction, and F4: residual fraction. Studies have demonstrated variability in the bioavailability of diverse forms of HMs. Among these, F1 and F2 were relatively unstable, highly bioavailable, and easily leached, whereas F3 and F4 exhibited relative stability and low bioavailability [28]. In addition, the risk assessment code (*RAC*) for HMs is often used to assess the environmental impact of F1 forms in sludge biochar. The calculation is displayed in Equation (6).

(6) $$RAC=\frac{F1}{F1+F2+F3+F4}\times 100\%$$

Based on the *RAC* method, the environmental risks of HMs were classified into five levels of hazard, as shown in Table 1.

## 3. Results and Discussion

### 3.1. Thermogravimetric Analysis of Sludge

**Figure 1:**
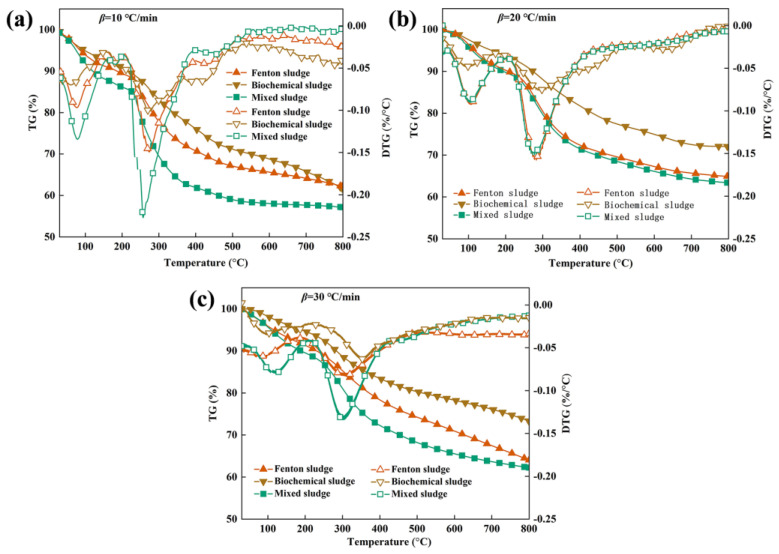
Thermogravimetry/Derived thermogravimetry (TG/DTG) curves of individual pyrolysis and co-pyrolysis of sludge at heating rates of 10 (**a**), 20 (**b**) and 30 (**c**) °C/min.

Figure 1 displays the TG/DTG curves of Fenton, biochemical, and mixed sludge at ramping rates of 10 °C/min, 20 °C/min, and 30 °C/min. The trend of sludge pyrolysis was essentially similar across different heating rates. The TG curves for the co-pyrolysis of mixed sludge exhibited a more prominent weight loss trend than those for individual sludge pyrolysis, indicating a higher weight loss. This phenomenon was further accentuated at a lower heating rate. From the TG curves, it is evident that the weight loss occurred in three stages, from room temperature to 800 °C, irrespective of whether the sludge was subjected to individual or co-pyrolysis. (1) For the initial phase, the primary phenomenon was the release of bound water when heating below 200 °C [29]. The moisture content of the sludge decreased after drying, resulting in a weight loss of only about 5%. (2) During the second stage, the temperature range of 200–500 °C was responsible for the decomposition of organic matter within the sludge. The breaking of chemical bonds between substances was the primary stage of weight loss, which ranged from 17% to 32%. The organic matter was retained in the form of fixed carbon and decomposed to produce reducing gases that facilitated the reduction of the metal oxides [30]. (3) During the third stage, the curve showed a slowdown in the decreasing trend once the temperatures exceeded 500 °C. The weight loss was approximately between 5% and 10%. This stage mostly involves the carbonization of residual hydrocarbons at high temperatures and the decomposition of inorganic substances that are difficult to degrade [31].

Based on the DTG curves, it is apparent that sludge pyrolysis resulted in two weight loss peaks, with the major one being the second peak. The weight loss peaks were correlated to various temperatures due to the variability of the components in the sludge. Significantly, the temperature peaks of the mixed sludge were consistently lower than those of the individual sludge. This suggested that co-pyrolysis of the mixed sludge promoted the decomposition of the sludge and advanced the reaction processes of the materials. Additionally, it was generally observed that the maximum weight loss rate was higher during co-pyrolysis compared to sludge pyrolysis conducted individually. This confirmed that co-pyrolysis helped to promote the decomposition of substances during the pyrolysis process. The maximum weight loss rate of Fenton sludge was higher than that of biochemical sludge, which was probably due to the positive impact of metal oxidizers of Fe and Al in Fenton sludge on sludge pyrolysis [32].

The heating rates had a significant effect on the pyrolysis of the sludge, as shown in Table 2. As the heating rates increased, the weight loss of the sludge decreased for both the individual pyrolysis and the co-pyrolysis. When the heating rates were raised from 10 to 30 °C/min, the maximum weight loss of Fenton sludge, biochemical sludge, and mixed sludge dropped from 37.84%, 38.26%, and 42.84% to 35.83%, 26.64%, and 37.75%, respectively. The incomplete reaction was possibly due to uneven heating caused by a higher heating rate of the materials [33]. In addition, the maximum weight loss of mixed sludge was higher than that of both Fenton sludge and biochemical sludge when the heating rates were increased from 10 to 30 °C/min, indicating that co-pyrolysis can increase the weight loss rate of pyrolysis. Meanwhile, the temperatures at the maximum weight loss rates for both individual and co-pyrolysis of sludge appeared to enlarge in different degrees as the heating rates increased. When the heating rate was 10 °C/min, the corresponding temperatures at the maximum weight loss rates of Fenton sludge, biochemical sludge, and mixed sludge were 274.31 °C, 275.62 °C, and 256.97 °C, respectively, which were reduced by 32.04 °C, 78.43 °C, and 47.44 °C compared with those at 30 °C/min.

The temperature of sludge decomposition moved into the high temperature range as the heating rates increased. This thermal hysteresis phenomenon was intensified with an increase in heating rates [34]. The impact of the heating rate on the maximum weight loss rates was consistent with the prior findings. The maximum weight loss rates of Fenton sludge, biochemical sludge, and mixed sludge decreased from 0.15 %/°C, 0.10 %/°C, and 0.23 %/°C to 0.08 %/°C, 0.06 %/°C, and 0.13 %/°C, respectively, as the pyrolysis heating rates were increased from 10 °C/min to 30 °C/min. Furthermore, pyrolysis on the surface of the substance occurred before pyrolysis in the internal part, leading to difficulty in the volatilization of gases produced by pyrolysis in the internal part. When the gases inside the material accumulate to a certain level, it inhibits the further decomposition of the material [35]. This not only induced a lower rate of weight loss but also discouraged the sludge pyrolysis reaction. The heating rate was set at 5 °C/min to achieve a more complete decomposition and precipitation of the materials.

### 3.2. Kinetic Reaction Process of Sludge Pyrolysis

**Figure 2:**
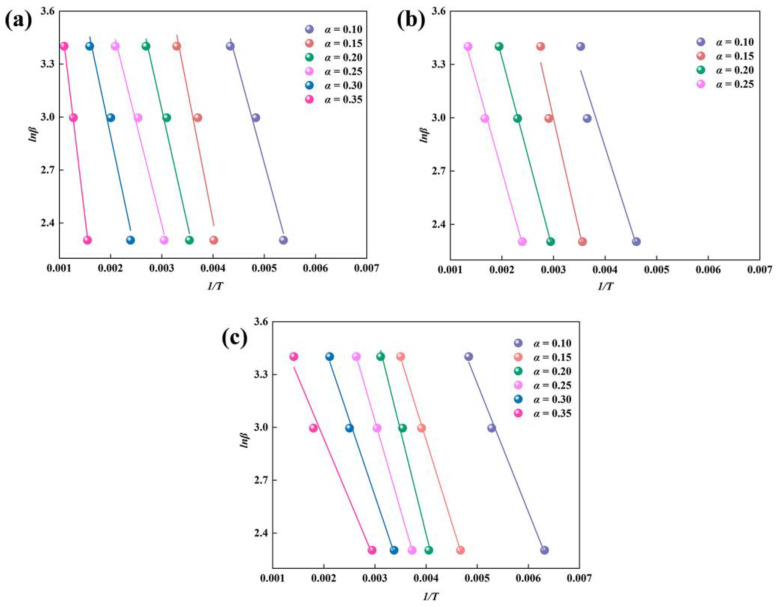
Fitted curves of Flynn-Wall-Ozawa (FWO) method for Fenton sludge (**a**), biochemical sludge (**b**), and mixed sludge (**c**).

The parameter *E_α_* is a crucial indicator for analyzing pyrolysis kinetics, as it reveals the reaction difficulties of substances during the pyrolysis process [36]. When the value of *E_α_* is lower, it indicates that the sludge pyrolysis process is more easily reactive. The data from thermogravimetric experiments were collected for Fenton sludge, biochemical sludge, and mixed sludge at heating rates of 10, 20, and 30 °C/min, respectively. The activation energies of Fenton sludge, biochemical sludge, and mixed sludge were calculated separately according to Equation (3) to clarify the effect of co-pyrolysis on sludge pyrolysis. The data of *lnβ* and *1*/*T* at *α* = 0.1–0.35 computed through the FWO method were linearly fitted by Origin (2022), and the results are illustrated in Figure 2.

The linear fitting equations and correlation coefficients (R^2^) for Fenton sludge, biochemical sludge, and mixed sludge were obtained from Figure 2, and the activation energy was calculated based on the slope of the equation *k* = −1.0516 *E_α_*/*R*. As shown in Table 3, the R^2^ values for Fenton sludge, biochemical sludge, and mixed sludge at various conversion rates ranged from 0.931 to 0.999, indicating a strong linear correlation. The average activation energies obtained according to the FWO method were 11.59 kJ/mol, 8.50 kJ/mol, and 7.11 kJ/mol, respectively. The average activation energy of the mixed sludge was lower than that of Fenton sludge and biochemical sludge, indicating that co-pyrolysis could reduce the reaction activation energy and facilitate the reaction process. Co-pyrolysis of Fenton sludge and biochemical sludge altered the pyrolysis reaction path and increased the reaction rate, resulting in lower activation energy during pyrolysis. It was also demonstrated that the co-pyrolysis of sludge can reduce the activation energy of the pyrolysis process through catalysis and adsorption [37,38]. Meanwhile, the temperature and energy required from the outside world were lower for the same degree of pyrolysis.

The slopes of the fitted equations for the same sludge at various conversion rates differed slightly due to the different substances reacting at each pyrolysis stage. The activation energy of the reaction was generally low in the initial stage, which was primarily attributed to the decomposition of the lighter substances. The higher reactivity precipitated small molecular hydrocarbons with reducing properties and highly reactive hydroxyl radicals (•OH), which was able to further promote the reactivity of other substances. The residual hydrocarbons and inorganic compounds in the sludge gradually decomposed as the temperature increased. The activation energy of the reaction was predominantly higher due to the relative stability and thermal durability of the chemical bonds that connect them. Consequently, Fenton sludge had an abundance of aromatic organics and metals, and its activation energy was higher than that of biochemical sludge.

### 3.3. Physical and Chemical Properties of Sludge and Sludge Biochar

The contents of major elements and HMs of both types of sludge were tested prior to pyrolysis, and the results are presented in Table 4. Comparing the two types of sludge, it is evident that the Fenton sludge contained less organic matter. The Fe and Al contents of the Fenton sludge exceeded those of the biochemical sludge, while the Pb and Zn contents of the biochemical sludge were higher.

The sludge biochars prepared from Fenton sludge, biochemical sludge, and mixed sludge at 800 °C pyrolysis were defined as FBC-800, BBC-800, and MBC-800, respectively. The yield, pH, elemental content, and specific surface area of FBC-800, BBC-800, and MBC-800 were listed in Table 5. The sludge contained significant amounts of metals and minerals, which were usually retained as solids in the biochar. The yield of sludge biochar from co-pyrolysis was higher in comparison to other types of biochar. Co-pyrolysis promoted the decomposition and precipitation of materials in the sludge, resulting in lower production of MBC-800 as opposed to FBC-800 and BBC-800. The large decomposition of organic acids during pyrolysis at high temperatures resulted in a neutral or alkaline pH of the biochar. The pH of the MBC-800 was significantly higher, possibly due to co-pyrolysis resulting in the generation of alkaline organic anions on the biochar surface. In particular, the natural alkalinity of biochar was conducive to enhancing the quality of acidic soils [39]. Compared with the raw materials, the contents of C and H in the sludge declined in different degrees after pyrolysis, suggesting that the organic matter in the sludge was decomposed in large quantities at high temperatures and released as CO and H_2_ gases. Both BBC-800 and MBC-800 demonstrated a high specific surface area attributable to the well-developed pore structure. In addition, the Fe and Al contents of MBC-800 were reduced by 10.43% and 5.63%, respectively, compared with that of FBC-800, which implied that the co-pyrolysis of sludge was able to reduce the metals in sludge.

### 3.4. Surface Morphology and Structural Alterations of Biochar

**Figure 3:**
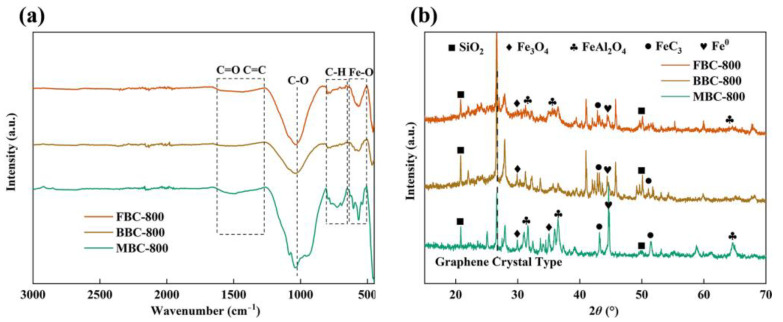
Fourier transform infrared (FTIR) spectroscopy (**a**) and X-ray diffraction (XRD) (**b**) of FBC-800, BBC-800, and MBC-800.

The functional groups on the surface of the three sludge biochars are demonstrated in Figure 3. The FTIR spectra of FBC-800, BBC-800, and MBC-800 exhibited minimal variation, suggesting that the functional groups had similar structures. The characteristic absorption peaks at 1610 cm^−1^~1350 cm^−1^ were caused by aromatic C=O and C=C stretching and vibration. The characteristic absorption peak at 1034 cm^−1^ was corresponding to C-O, and around 793 cm^−1^ was classified as C-H in the aromatic group. In particular, the characteristic absorption peak at 560 cm^−1^ was attributable to the Fe-O bond. The surface of the sludge biochar contained various oxygen-containing functional groups, while Fe was loaded onto the biochar surface in the form of oxides. Compared with BBC-800, the intensities of the absorption peaks of FBC-800 and MBC-800 were clearly enhanced, which was attributed to the fact that FBC-800 was Al modified to benefit the retention of functional groups in biochar [40]. The MBC-800 generated more functional group structures because co-pyrolysis promoted further polycondensation and aromatization of organic matter [41].

Comparing standard reference cards with XRD patterns associated with iron phase species allows further analysis of crystal particle composition. Figure 3 showed the appearance of the characteristic diffraction peaks of FeAl_2_O_4_ at 2θ of 31.66°, 36.0°, and 64.6° in FBC-800 and MBC-800, corresponding to crystal planes of (220), (311), and (440) (JCPDS No. 01-071-4915), respectively. The crystal planes of (220) and (311) (JCPDS No. 72-2303) corresponded to the diffraction peaks of Fe_3_O_4_ at 2θ of 30.00° and 35.52°, respectively. Additionally, the diffraction peaks of FeC_3_ were observed at 2θ of 43.18° and 51.42° (JCPDS No. 35-0772). Notably, the crystal surface of Fe0(110) (JCPDS No. 06-0696) was observed at 2θ of 44.66° (JCPDS No. 06-0696), and the production of low-valence iron suggested that reducing gases were generated during pyrolysis. There were few variations observed in the phase composition due to the resemblance of the sludge constituents. The low content of Al in the biochemical sludge and the absence of FeAl_2_O_4_ in the XRD pattern suggested that fewer metal active sites were involved in the reaction.

**Figure 4:**
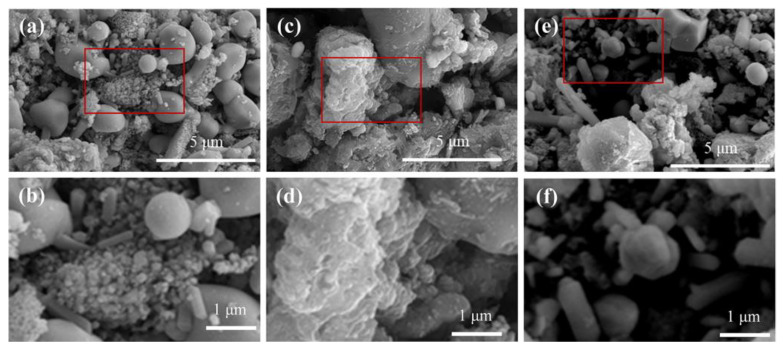
Morphological observations of FBC-800 (**a**,**b**), BBC-800 (**c**,**d**), and MBC-800 (**e**,**f**) by the scanning electron microscopy (SEM). (**b**,**d**,**f**) are the red squares of (**a**,**c**,**e**), respectively.

The microscopic morphology of FBC-800, BBC-800, and MBC-800 was shown in the SEM image in Figure 4. Owing to the carbonization of the organic matter in sludge, the biochar developed a rough surface structure [42]. As shown in Figure 4, the surface morphology of the three sludge biochars exhibited significant differences. A large amount of particulate matter accumulated on the surface of FBC-800 with little effective pore structure observed, which may also be attributed to its comparatively small specific surface area. On the contrary, the BBC-800 had a well-developed pore structure and an apparent layered sheet-like structure that served as the skeleton of the biochar, which was composed of carbon and hydrogen as its primary constituents. The surface of MBC-800 was loaded with various particle structures of different shapes, and MBC-800 still retained a certain degree of pore structure.

### 3.5. Role of Co-Pyrolysis on the Immobilization of HMs in Sludge

**Figure 5:**
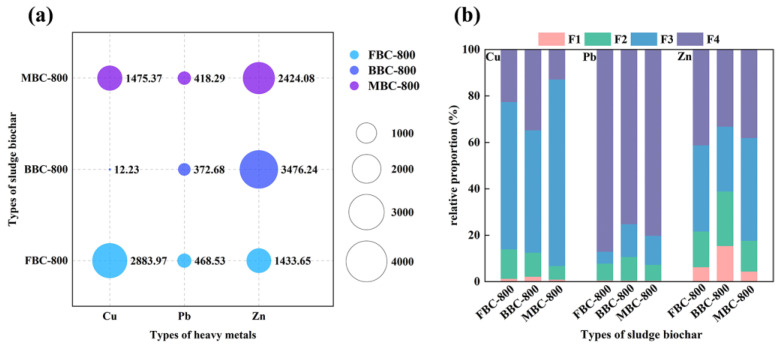
The contents (**a**) and morphological distribution (**b**) of Cu, Pb, and Zn in FBC-800, BBC-800, and MBC-800.

Cu, Pb, and Zn are common HMs in sludge. The presence of high levels of HMs is not only a threat to aquatic organisms but also a significant health hazard to humans [43]. The potential for high-value utilization of sludge biochar is key to the evaluation of sludge pyrolysis technologies, thus placing a higher demand on the application of sludge biochar as a resource. Consequently, the study of the immobilization and bioavailability of HMs in sludge biochar is necessary [44]. Table 4 displays the concentrations of HMs in Fenton sludge and biochemical sludge. The contents of Cu, Pb, and Zn in the mixed sludge were 1037.12 mg/kg, 316.86 mg/kg, and 1748.15 mg/kg, respectively. It was shown in Figure 5 that the total content of Cu, Pb, and Zn and the distribution of different forms of the sludge biochar prepared by individual pyrolysis and co-pyrolysis.

The contents of Cu in FBC-800, BBC-800, and MBC-800 were 2883.97 mg/kg, 12.23 mg/kg, and 1475.37 mg/kg, respectively, as shown in Figure 5. In addition, the immobilization rates, which were calculated from Equation (5), were 85.74%, 82.67%, and 85.84% for the corresponding materials. The contents of Pb in FBC-800, BBC-800, and MBC-800 were 468.53 mg/kg, 372.68 mg/kg, and 418.29 mg/kg, respectively, with immobilization rates of 76.98%, 71.47%, and 79.66%. Given that Pb is a volatile HM, the sludge pyrolysis process had a low immobilization rate of Pb [45]. The contents of Zn in FBC-800, BBC-800, and MBC-800 were 1433.65 mg/kg, 3476.24 mg/kg, and 2424.08 mg/kg, respectively, and the immobilization rates were 80.16%, 71.19%, and 83.67% in the successive order.

Based on the above results, it was found that the immobilization rate of HMs after sludge pyrolysis treatment was higher than 70%, meaning that most of the HMs were immobilized in the biochar. A comparison of the immobilization rates of HMs with different biochars indicated that MBC-800 had higher immobilization rates for Cu, Pb, and Zn than FBC-800 and BBC-800. In particular, MBC-800 showed significant differences compared to BBC-800 (*p* < 0.05). Therefore, co-pyrolysis enhanced the immobilization of HMs and diminished the impact of possible secondary contamination due to the volatilization of HMs. Furthermore, the contents of Cu, Pb, and Zn after co-pyrolysis were found to be below the limit values established by the “Pollutant Control Standards for Agricultural Sludge” (GB4284-2018), which were 1500 mg/kg for Cu, 1000 mg/kg for Pb, and 3000 mg/kg for Zn. This indicated that co-pyrolysis had the potential to reduce the toxicity of certain high-concentration HMs present in the single sludge and further enhance the possibility of sludge resource utilization.

### 3.6. Environmental Risk Assessment of HMs

As shown in Figure 5, the primary existing forms of Cu in FBC-800, BBC-800, and MBC-800 were F3. Among these, the proportions of F1 in the bioavailability were 1.23%, 2.12%, and 0.88%, respectively, while those of F2 were 12.68%, 10.38%, and 5.87%. The risk evaluation of Cu in MBC-800 only was none, indicating the lowest possible bioavailability of Cu in MBC-800. It also validated the fact that co-pyrolysis improved the immobilization of HMs in another way. The major existing forms of Pb in FBC-800, BBC-800, and MBC-800 were all F4, which could be attributed to the increases in pH of the biochar produced through pyrolysis. Additionally, the Pb existed in the form of hydroxides and was immobilized in the residue as insoluble oxides during the extraction process [46]. The percentages of F1 in its bioavailability were 0.52%, 0.43%, and 0.36%, whereas F2 accounted for 7.22%, 10.22%, and 6.87%, respectively. It was slightly different from the case of immobilization for Cu, as Pb in the acid-soluble state was hardly detected in the sludge biochar after the pyrolysis treatment. Consequently, the risk index evaluations of Pb were all none, and Pb had the lowest bioavailability in MBC-800. The main existing forms of Zn in FBC-800, BBC-800, and MBC-800 were F3 and F4. The proportions of F1 in the bioavailability were 6.25%, 15.36%, and 4.36%, and for F2, they were 15.37%, 23.5%, and 13.25%, respectively. Compared to Cu and Pb, Zn showed a slightly higher bioavailability. It had also been proposed that Zn exhibited less stability during pyrolysis as it mainly existed interactively in the form of ZnO, ZnSiO_3_, and Zn. The co-pyrolysis still had a significant effect on reducing the bioavailability of Zn, which was probably related to the enhancement of the surface functional group activity of the sludge after pyrolysis, resulting in an effective reduction of the environmental risk of HMs in sludge biochar. Studies conducted on the leaching toxicity of HMs by pyrolyzing municipal sludge also showed that pyrolysis can inhibit the leaching of HMs from sludge [47].

## 4. Conclusions

This study revealed the pyrolysis characteristics and kinetic analysis of Fenton sludge and biochemical sludge, elucidated the physicochemical properties and morphological structure of sludge biochar, and emphasized that co-pyrolysis effectively reduced the environmental risk of HMs in sludge. The co-pyrolysis of Fenton sludge and biochemical sludge showed the advantages of enhancing weight loss and weight loss rates of sludge pyrolysis and reducing decomposition temperature at different heating rates. According to the FWO method, the activation energies for Fenton sludge, biochemical sludge, and mixed sludge were found to be 11.59 kJ/mol, 8.50 kJ/mol, and 7.11 kJ/mol, respectively. These results indicated that co-pyrolysis was able to reduce the activation energy of the reaction, leading to an enhancement in pyrolysis efficiency. This was important for the efficient reduction of sludge and the saving of treatment costs. Moreover, co-pyrolysis facilitated reactive processes in the sludge, promoting polycondensation and aromatization of organic matter while also creating a stable and porous biochar structure. The biochar produced from the co-pyrolysis of sludge had a higher pH and possessed an abundance of porous structures, surface functional groups, and diverse iron phase crystals, indicating its potential applications as a soil amendment and catalyst. Sludge biochar has the potential to improve water permeability and water retention by improving the physical structure of the soil and to facilitate the catalytic process of pollutants by increasing the active sites. Notably, the decrease in the bioavailability of HMs in sludge biochar following pyrolysis of the sludge had a significant effect on the reduction of toxicity from HMs. Compared to FBC-800 and BBC-800, MBC-800 showed higher immobilization rates for Cu, Pb, and Zn and significantly increased the percentages of F3 and F4, resulting in an effective reduction of environmental risks associated with HMs in sludge. In summary, this study revealed that co-pyrolysis was beneficial to enhance the immobilization of HMs in sludge, which provided an important theoretical basis for the resource utilization of sludge biochar and the mitigation of the toxicity of HMs.

## Figures and Tables

**Table 1:** Risk assessment grading of the morphology of HMs.

Level of Evaluation	Exponential Value
None	*RAC* ≤ 1%
Low	1% < *RAC* ≤ 10%
Moderate	10% < *RAC* ≤ 30%
High	30% < *RAC* ≤ 50%
Very high	*RAC* > 50%

**Table 2:** Maximum weight loss and maximum rate of weight loss of sludge in individual pyrolysis and co-pyrolysis at different heating rates.

Types of Sludge	Heating Rates (°C/min)	Maximum Weight Loss (%)	Temperatures at Maximum Weight Loss Rates (°C)	Maximum Weight Loss Rates (%/°C)
Fenton Sludge	30	35.83	306.35	0.08
	20	35.79	282.64	0.16
	10	37.84	274.31	0.15
Biochemical Sludge	30	26.64	354.05	0.06
	20	27.95	302.69	0.08
	10	38.26	275.62	0.10
Mixed Sludge	30	37.75	304.41	0.13
	20	37.00	277.90	0.15
	10	42.84	256.97	0.23

**Table 3:** The activation energy of Fenton sludge, biochemical sludge, and mixed sludge.

Types of Sludge	*α*	FWO			
		Fitted Equations	R^2^	*E_α_* (kJ/mol)	*E* (kJ/mol)
Fenton Sludge	0.10	y = −1062.54x + 8.05	0.984	8.40	11.59
	0.15	y = −1489.45x + 8.37	0.948	11.78	
	0.20	y = −1299.17x + 6.94	0.985	10.27	
	0.25	y = −1156.67x + 5.86	0.987	9.14	
	0.30	y = −1372.51x + 5.64	0.972	10.85	
	0.35	y = −2415.59x + 6.06	0.999	19.10	
Biochemical Sludge	0.10	y = −907.84x + 6.47	0.931	7.18	8.50
	0.15	y = −1269.57x + 6.80	0.965	10.04	
	0.20	y = −1094.91x + 5.53	0.999	8.66	
	0.25	y = −1028.66x + 4.78	0.996	8.13	
Mixed Sludge	0.10	y = −728.21x + 6.89	0.995	5.76	7.11
	0.15	y = −935.29x + 6.67	0.999	7.39	
	0.20	y = −1172.40x + 7.08	0.990	9.27	
	0.25	y = −1009.68x + 6.07	0.999	7.98	
	0.30	y = −860.46x + 5.19	0.995	6.80	
	0.35	y = −691.75x + 4.32	0.983	5.47	

**Table 4:** The content of major elements and heavy metals (HMs) in Fenton sludge and biochemical sludge.

Sample	Major Elements (%)					HMs (mg/kg)		
	C	H	Si	Fe	Al	Cu	Pb	Zn
Fenton Sludge	12.01	2.67	8.13	23.67	8.21	2158.32	372.41	1094.34
Biochemical Sludge	27.14	4.23	8.88	5.36	0.54	10.15	357.76	3350.14

**Table 5:** Physicochemical properties of sludge biochar.

Types of Biochar	Yield (%)	pH	Contents of Elements (%)					Specific Surface Area (m^2^/g)
			C	H	Si	Fe	Al	
FBC-800	61.19	7.76	2.16	0.34	14.12	29.36	11.54	7.62
BBC-800	68.61	6.21	6.37	0.85	15.73	10.06	1.48	64.23
MBC-800	60.34	8.67	4.56	0.49	16.18	18.93	5.91	48.23

## Data Availability

Information is available in the manuscript.

## References

[1] Caligan C.J.A., Garcia M.M.S., Mitra J.L., San Juan J.L.G. (2022). Multi-objective optimization for a wastewater treatment plant and sludge-to-energy network. J. Clean. Prod..

[2] Duan B., Feng Q. (2021). Comparison of the potential ecological and human health risks of heavy metals from sewage sludge and livestock manure for agricultural use. Toxics.

[3] Li M., Wang H., Huang Z., Yuan X., Tan M., Jiang L., Wu Z., Qin X., Li H. (2020). Comparison of atmospheric pressure and gas-pressurized torrefaction of municipal sewage sludge: Properties of solid products. Energy Conv. Manag..

[4] Li D., Shan R., Jiang L., Gu J., Zhang Y., Yuan H., Chen Y. (2022). A review on the migration and transformation of heavy metals in the process of sludge pyrolysis. Resour. Conserv. Recycl..

[5] Soria-Verdugo A., Morato-Godino A., Garcia-Gutierrez L.M., Garcia-Hernando N. (2017). Pyrolysis of sewage sludge in a fixed and a bubbling fluidized bed—Estimation and experimental validation of the pyrolysis time. Energy Convers. Manag..

[6] Wu M., Yang Z.-H., Jiang T.-B., Zhang W.-W., Wang Z.-W., Hou Q.-X. (2023). Enhancing sludge methanogenesis with changed micro-environment of anaerobic microorganisms by Fenton iron mud. Chemosphere.

[7] Chi B., Huang Y., Xiong Z., Tan J., Zhou W., Yang Z., Zhou K., Duan X., Chen A., Zha R. (2023). Sludge lysis by thermophilic bacteria community enhances nutrient removal, sludge reduction, and modulates microbial community in anaerobic-anoxic-oxic process. J. Water Process. Eng..

[8] Chae J.-S., Choi S.-A., Kim Y.-H., Oh S.-C., Ryu C.-K., Ohm T.-I. (2016). Experimental study of fry-drying and melting system for industrial wastewater sludge. J. Hazard. Mater..

[9] Xu Z.-X., Song H., Deng X.-Q., Zhang Y.-Y., Xue-Qin M., Tong S.-Q., He Z.-X., Wang Q., Shao Y.-W., Hu X. (2019). Dewatering of sewage sludge via thermal hydrolysis with ammonia-treated Fenton iron sludge as skeleton material. J. Hazard. Mater..

[10] Wang M., Sun Y., Yu Q., Zhao Z., Li Y., Zhang Y. (2023). Sustainable disposal of Fenton sludge and enhanced organics degradation based on dissimilatory iron reduction in the hydrolytic acidification process. J. Hazard. Mater..

[11] Shen M., Huang Z., Luo X., Ma Y., Chen C., Chen X., Cui L. (2020). Activation of persulfate for tetracycline degradation using the catalyst regenerated from Fenton sludge containing heavy metal: Synergistic effect of Cu for catalysis. Chem. Eng. J..

[12] Gao N., Kamran K., Quan C., Williams P.T. (2020). Thermochemical conversion of sewage sludge: A critical review. Prog. Energy Combust. Sci..

[13] Xing X., Zhao H., Zhou L., Wang Y., Chen H., Gao Y., Wang Y., Zhu Y. (2022). Pyrolysis kinetics, thermodynamics of PTA sludge and product characterization of cyclic in-situ catalytic pyrolysis by using recycled char as a catalyst. Energy.

[14] Jiang G., Xu D., Hao B., Liu L., Wang S., Wu Z. (2021). Thermochemical methods for the treatment of municipal sludge. J. Clean. Prod..

[15] Long X., Zhang R., Rong R., Wu P., Chen S., Ao J., An L., Fu Y., Xie H. (2023). Adsorption characteristics of heavy metals Pb^2+^ and Zn^2+^ by magnetic biochar obtained from modified AMD sludge. Toxics.

[16] Yang Y., Luo X., Zhang J., Ma X., Sun P., Zhao L. (2022). Sewage sludge–coconut fiber co-pyrolysis biochar: Mechanisms underlying synergistic heavy metal stabilization and ciprofloxacin adsorption. J. Clean. Prod..

[17] Li Z., Yu D., Liu X., Wang Y. (2023). The fate of heavy metals and risk assessment of heavy metal in pyrolysis coupling with acid washing treatment for sewage sludge. Toxics.

[18] Alharbi H.A., Alotaibi K.D., El-Saeid M.H., Giesy J.P. (2023). Polycyclic aromatic hydrocarbons (PAHs) and metals in diverse biochar products: Effect of feedstock type and pyrolysis temperature. Toxics.

[19] Wang F., Zhang S., Cheng P., Zhang S., Sun Y. (2020). Effects of soil amendments on heavy metal immobilization and accumulation by maize grown in a multiple-metal-contaminated soil and their potential for safe crop production. Toxics.

[20] Liu L., Liu X., Wang D., Lin H., Huang L. (2020). Removal and reduction of Cr(VI) in simulated wastewater using magnetic biochar prepared by co-pyrolysis of nano-zero-valent iron and sewage sludge. J. Clean. Prod..

[21] Khan R., Shukla S., Kumar M., Zuorro A., Pandey A. (2023). Sewage sludge derived biochar and its potential for sustainable environment in circular economy: Advantages and challenges. Chem. Eng. J..

[22] Mohamed B.A., Ruan R., Bilal M., Khan N.A., Awasthi M.K., Amer M.A., Leng L., Hamouda M.A., Vo D.V.N., Li J. (2023). Co-pyrolysis of sewage sludge and biomass for stabilizing heavy metals and reducing biochar toxicity: A review. Environ. Chem. Lett..

[23] Chen X., Ma R., Luo J., Huang W., Fang L., Sun S., Lin J. (2021). Co-microwave pyrolysis of electroplating sludge and municipal sewage sludge to synergistically improve the immobilization of high-concentration heavy metals and an analysis of the mechanism. J. Hazard. Mater..

[24] Das A., Dey A. (2020). P-Nitrophenol -Bioremediation using potent Pseudomonas strain from the textile dye industry effluent. J. Environ. Chem. Eng..

[25] Li Y., Li Y., Zhang Z., He X., Chen J., Liu C. (2022). Pyrolysis kinetics of manganese carbonate. J. Therm. Anal. Calorim..

[26] Urych B., Smoliński A. (2016). Kinetics of sewage sludge pyrolysis and air gasification of its chars. Energy Fuels.

[27] Li C., Li J., Xie S., Zhang G., Pan L., Wang R., Wang G., Pan X., Wang Y., Angelidaki I. (2022). Enhancement of heavy metal immobilization in sewage sludge biochar by combining alkaline hydrothermal treatment and pyrolysis. J. Clean. Prod..

[28] Zhang S., Gu W., Geng Z., Bai J., Dong B., Zhao J., Zhuang X., Shih K. (2023). Immobilization of heavy metals in biochar by co-pyrolysis of sludge and CaSiO_3_. J. Environ. Manag..

[29] Li J., Hu J., Wang T., Gan J., Xie J., Shui Y., Liu J., Xue Y. (2019). Thermogravimetric analysis of the co-combustion of residual petrochemical sludge and municipal sewage sludge. Thermochim. Acta.

[30] Guo Z., Bai G., Huang B., Cai N., Guo P., Chen L. (2021). Preparation and application of a novel biochar-supported red mud catalyst: Active sites and catalytic mechanism. J. Hazard. Mater..

[31] Magdziarz A., Werle S. (2014). Analysis of the combustion and pyrolysis of dried sewage sludge by TGA and MS. Waste Manag..

[32] Yang J., Xu X., Liang S., Guan R., Li H., Chen Y., Liu B., Song J., Yu W., Xiao K. (2018). Enhanced hydrogen production in catalytic pyrolysis of sewage sludge by red mud: Thermogravimetric kinetic analysis and pyrolysis characteristics. Int. J. Hydrogen Energy.

[33] Li Q., Yang H., Chen P., Jiang W., Chen F., Yu X., Su G. (2023). Investigation of catalytic co-pyrolysis characteristics and synergistic effect of oily sludge and walnut shell. Int. J. Environ. Res. Public Health.

[34] Ma Z., Xie J., Gao N., Quan C. (2019). Pyrolysis behaviors of oilfield sludge based on Py-GC/MS and DAEM kinetics analysis. J. Energy Inst..

[35] Xu G., Cai X., Wang L., Zhang Q., Fang B., Zhong X., Yao J. (2022). Thermogravimetric-infrared analysis and performance optimization of co-pyrolysis of oily sludge and rice husks. Int. J. Hydrogen Energy.

[36] Zhang M., He T., Jin B. (2023). Effect of mineral additives on pyrolytic characteristics and heavy metal behavior during co-pyrolysis of industrial sludge and hyperaccumulator plant. J. Anal. Appl. Pyrolysis.

[37] Fang S., Yu Z., Ma X., Lin Y., Chen L., Liao Y. (2018). Analysis of catalytic pyrolysis of municipal solid waste and paper sludge using TG-FTIR, Py-GC/MS and DAEM (distributed activation energy model). Energy.

[38] Yang K., Sun J., Liu H., Yang W., Dong L. (2023). Study on the Thermogravimetric Kinetics of Dehydrated Sewage Sludge Regulated by Cationic Polyacrylamide and Sawdust. Polymers.

[39] Yuan H., Lu T., Huang H., Zhao D., Kobayashi N., Chen Y. (2015). Influence of pyrolysis temperature on physical and chemical properties of biochar made from sewage sludge. J. Anal. Appl. Pyrolysis.

[40] Fan J., Li Y., Yu H., Li Y., Yuan Q., Xiao H., Li F., Pan B. (2020). Using sewage sludge with high ash content for biochar production and Cu(II) sorption. Sci. Total Environ..

[41] Wang J., Wang T., Zhu Q., Zhang S., Shi Q., Chovelon J.-M., Wang H. (2021). Preparation of a novel sludge-derived biochar by K_2_FeO_4_ conditioning to enhance the removal of Pb^2+^. Colloid Interface Sci. Commun..

[42] Shen M., Huang Z., Qiu L., Chen Z., Xiao X., Mo X., Cui L. (2020). Recycling of Fenton sludge containing Ni as an efficient catalyst for tetracycline degradation through peroxymonosulfate activation. J. Clean. Prod..

[43] Sultana S., Hossain M.B., Choudhury T.R., Yu J., Rana M.S., Noman M.A., Hosen M.M., Paray B.A., Arai T. (2022). Ecological and human health risk assessment of heavy metals in cultured shrimp and aquaculture sludge. Toxics.

[44] Fan Z., Zhou X., Peng Z., Wan S., Gao Z.F., Deng S., Tong L., Han W., Chen X. (2023). Co-pyrolysis technology for enhancing the functionality of sewage sludge biochar and immobilizing heavy metals. Chemosphere.

[45] Chanaka Udayanga W.D., Veksha A., Giannis A., Lisak G., Chang V.W.C., Lim T.-T. (2018). Fate and distribution of heavy metals during thermal processing of sewage sludge. Fuel.

[46] Song F., Gu L., Zhu N., Yuan H. (2013). Leaching behavior of heavy metals from sewage sludge solidified by cement-based binders. Chemosphere.

[47] Zhou A., Yu S., Deng S., Mikulčić H., Tan H., Wang X. (2023). Enrichment characteristics and environmental risk assessment of heavy metals in municipal sludge pyrolysis biochar. J. Energy Inst..

